# Electrophilic Properties of 2′-Deoxyadenosine···Thymine Dimer: Photoelectron Spectroscopy and DFT Studies

**DOI:** 10.1021/acs.jpca.1c03803

**Published:** 2021-07-26

**Authors:** Piotr Storoniak, Janusz Rak, Haopeng Wang, Yeon Jae Ko, Kit H. Bowen

**Affiliations:** † Faculty of Chemistry, University of Gdańsk, Wita Stwosza 63, Gdańsk 80-308, Poland; ‡ Department of Chemistry, Johns Hopkins University, Baltimore, Maryland 21218, United States

## Abstract

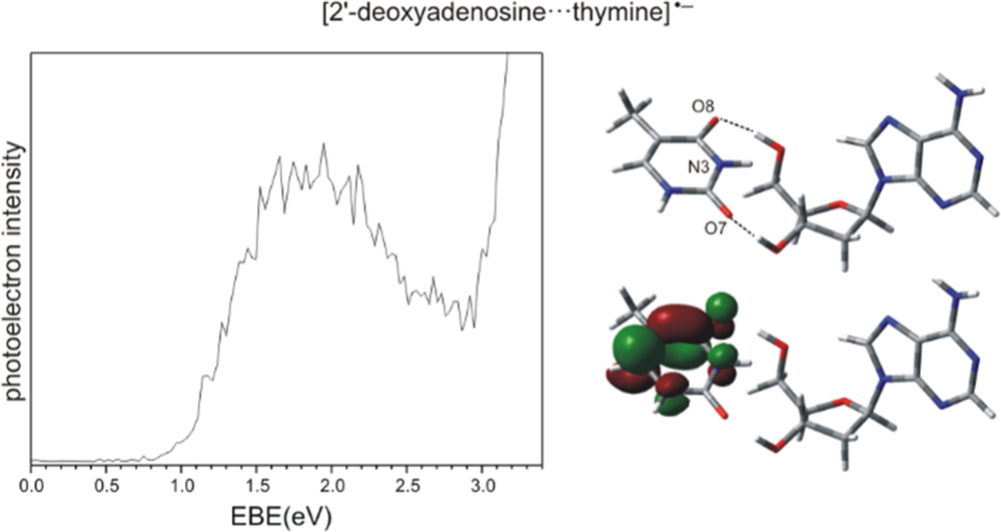

The anion radical of the 2′-deoxyadenosine···thymine (dAT^•–^) pair has been investigated experimentally and theoretically in the gas phase. By employing negative-ion photoelectron spectroscopy (PES), we have registered a spectrum typical for the valence-bound anion, featuring a broad peak at the electron-binding energy (EBE) between ∼1.5 and 2.2 eV with the maximum at ∼1.9 eV. The measured value of the adiabatic electron affinity (AEA) for dAT was estimated to be ∼1.1 eV. Calculations performed at the M06-2X/6-31++G(d,p) level revealed that the structure, where thymine is coordinated to the sugar of dA by two hydrogen bonds, is responsible for the observed PES signal. The AEA_G_ and the vertical detachment energy of 0.91 and 1.68 eV, respectively, calculated for this structure reproduce the experimental values well. The role of the possible proton transfer in the stabilization of anionic radical complexes is discussed.

## 1 Introduction

Unflagging interest in the electrophilic properties of DNA components can be assigned to the fact that low-energy electrons (LEEs—electrons with energies below 20 eV) are capable of inducing damage to this biopolymer under ultra-high vacuum (UHV).^1^ Experiments on the bombardment of isolated plasmid DNA with LEEs under UHV revealed a resonant character of interactions between electrons and DNA that lead to single- and double-strand breaks.^2−5^ LEE-induced damage was proposed to occur through dissociative electron attachment (DEA), which involves the formation of transient negative ions in the DNA molecule. A number of experimental and computational works have been devoted to the details of the DEA mechanisms of the DNA fragmentation upon LEE attachment.^6−9^ Resonance anionic states, short-living in the gas phase (e.g., π-shape resonances with lifetimes in the range of 10^–15^ to 10^–10^ s),^9^ instead of decomposition may undergo relaxation and conversion into stable valence-bound (VB) radical anions when interacting with the surrounding molecules.^9^ Formation of such valence anions of nucleobases is documented experimentally, and it has been found that they may be further stabilized by protonation at N, O, or C sites from proton donors, such as water molecules.^10^

Proton transfer (PT) seems to be a phenomenon of significant importance for the interaction of LEEs with DNA. Indeed, some results suggest that the neutralization of the excess charge by electron-induced PT may prevent DNA strands against cleavage.^9^ Molecular dynamics/density functional theory calculations for nucleotides H-bonded to water molecules demonstrated that the protonation of anion radical nucleotides 3′-dTMPH and 3′-dCMPH increases the barriers for the sugar–phosphate bond and glycosidic bond cleavages.^11^ On the other hand, the protonation of nucleobase anion radicals may produce reactive neutral radicals and introduce alterations to nucleobases, which cause harm to DNA.^12^

Formation of the electronically stable anionic states of nucleobases has became the main assumption in numerous theoretical studies on the role of LEEs in such destructive to DNA phenomena such as the sugar–phosphate bond cleavage^11,13−28^ and *N*-glycosidic bond cleavage.^11,21,25,26,28−32^ Extensive reviews devoted to the interactions of electrons with DNA components are available.^9,33−35^ Barrierless electron-induced PT was shown in the past to contribute to the stabilization of gaseous anion radical complexes involving nucleobases. Those studies revealed that both anionic nucleic bases, adenine and thymine, may be protonated as a result of the interaction with species of appropriate acidity, and adenine may also become a proton donor utilizing its N9H site or N6H site.^36^

**Scheme 1:**
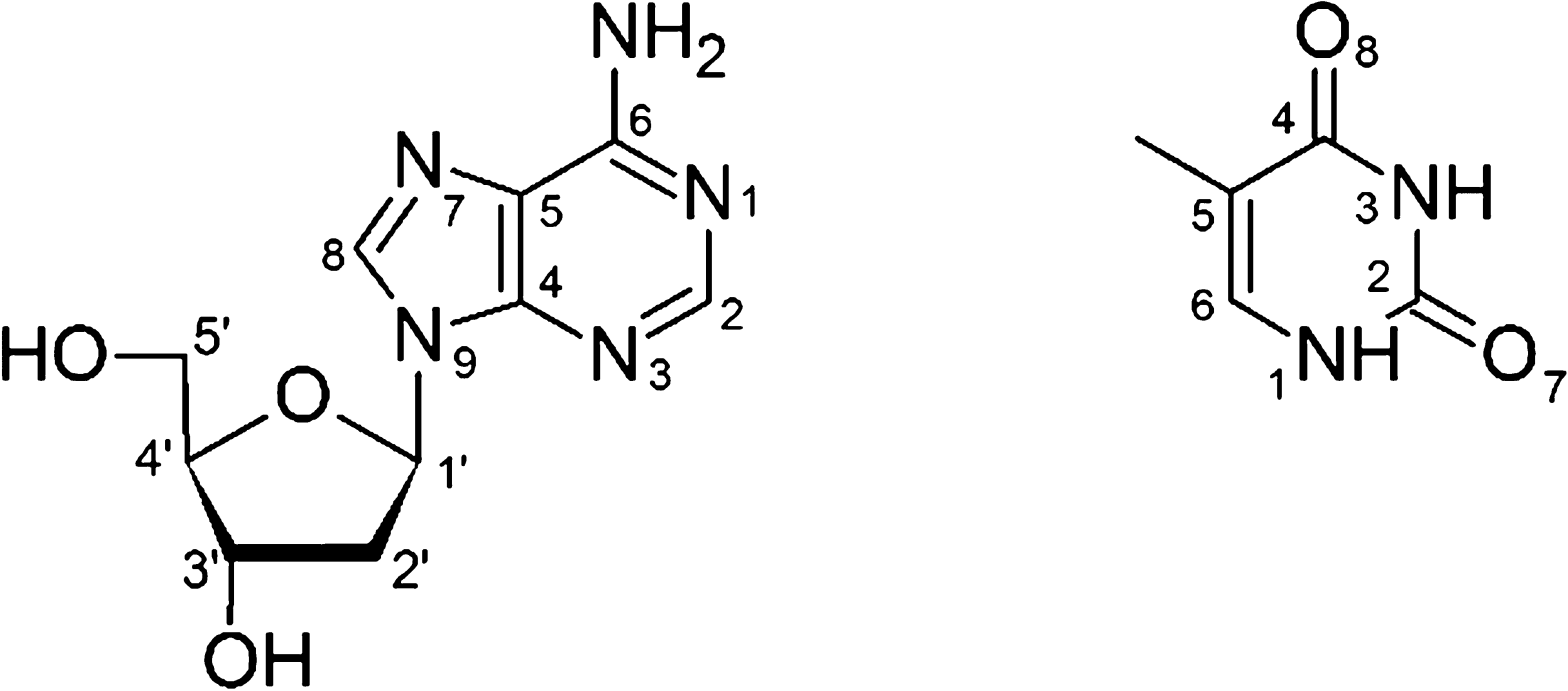
Chemical Structures of 2′-Deoxyadenosine (dA) and Thymine (T)

The present report is devoted to studies on the attachment of excess electrons to DNA building blocks. Namely, by means of pulsed laser infrared desorption, we introduced a nucleoside, 2′-deoxyadenosine (dA), and a nucleobase, thymine (T), into the gas phase (for the chemical structure of monomers and atom numbering, see Scheme 1). After the subsequent attaching of electrons to the gaseous mixture of monomers, we have observed using photoelectron spectroscopy (PES) that T and dA form a stable VB anion radical dimer dAT^•–^. Successful generation of gaseous dAT^•–^ in the PES experiment allowed for the determination of electron affinities and vertical detachment energy (VDE). Experimental results were supplemented by quantum chemical calculations, which provided details about the system under study. Thus, the computational part of the project allowed us to discuss the geometrical features and thermodynamic characteristics of the gaseous anionic dimer, as well as the excess electron localization within dAT^•–^ and significance of the PT phenomenon for the stabilization of this anion radical complex.

## 2 Methods

### 2.1 Experimental Methods

A mixture of neutral compounds, 2′-deoxyadenosine and thymine, covering a slowly translating graphite rod, was desorbed into the gas phase by the pulses of low-power infrared photons (1.17 eV/photon) from a Nd:YAG laser. Desorption of the molecules was caused by a rapid temperature rise of the graphite bar. Simultaneously, electrons were generated by visible laser pulses (another Nd:YAG laser operated at 532 nm, 2.33 eV/photon) striking a rotating yttrium oxide disk. Because yttrium oxide’s work function of ∼2 eV is slightly below the photon energy of the visible laser, LEEs were released into the gas phase. At the same time, a pulsed valve provided a collisionally cooled jet of helium to carry away excess energy and stabilize the resulting parent radical anions.

Subsequently, dAT^•–^ parent anions were extracted into a linear time-of-flight mass spectrometer (mass resolution ∼600), mass selected and crossed with a fixed frequency photon beam (a third Nd:YAG laser that operated at 355 nm, 3.49 eV/photon). The photodetached electrons were energy analyzed using a magnetic bottle energy analyzer with a resolution of 35 meV at EKE = 1 eV. Spectral bands in the photoelectron spectrum result from the vertical Franck–Condon overlap between the wave function of the anion and the wave function of the resulting neutral. This technique is based on the energy conserving relationship *h*ν = EBE + EKE, where *h*ν is the photon energy, EBE is the electron-binding energy of the observed transitions, and EKE is the measured kinetic energy of detached electrons. The EBE corresponding to the intensity maximum on the spectrum is referred to as the VDE.

Photoelectron spectra were calibrated against the well-known spectrum of Cu^–^. The details of a photoelectron spectrometer have been described previously.^37^

### 2.2 Computational Methods

In order to identify the geometry of the anion radicals, which are formed in the photoelectron experiment, we have performed calculations considering possible combinations of 2′-deoxyadenosine and bare thymine. Our computational approach is based on the assumption that the structure responsible for the signal in the photoelectron spectra should be thermodynamically equilibrated. Hence, based on the relative stabilities of particular anionic geometries in terms of Gibbs free energy, we should identify the structure responsible for the experimental picture. Adiabatic electron affinity (AEA) and VDE calculated for the most stable structure should coincide with the experimentally determined AEA and VDE values. The AEA can be defined in terms of Gibbs free energies, AEA_G_, as the difference between the Gibbs free energies of the neutral and the anion at their fully relaxed geometries: AEA_G_ = *G*(neu@neu) – *G*(an@an). In turn, VDE is the energy required for the detachment of an electron from an anion and corresponds to the difference between the absolute energies of the neutral and the anion, both at the optimized anion geometry: VDE = *E*(neu@an) – *G*(an@an). Positive VDE indicates that the energy of an anion is lower than the energy of the neutral and that the anion is stable against vertical electron autodetachment.

In this study, we have applied the density functional theory with the hybrid metafunctional M06-2X^38^ in combination with the 6-31++G(d,p) basis set.^39^ The M06-2X functional seems to be better suited for the description of noncovalent, dispersive-type interactions such as van der Waals attraction and π–π interaction^38,40^ than the B3LYP one and was shown to be successful in predicting the binding energies of hydrogen-bonded complexes^38,41^ as well as thermochemical properties.^38,40^ The benchmark study by Chen et al.^42^ demonstrated that M06-2X is suitable for modeling interactions between electrons and nucleotides and for predicting the kinetic barriers of the phosphodiester and glycosidic bond cleavages in the formed nucleotide anions. More recently, Borioni et al. addressed the performance of 23 DFT functionals and have concluded that M06 and B3PW91 are the best functionals for studying anions of organic compounds.^43^ In another study, the comparison of the M06X results with the high-level complete-active-space self-consistent field second-order perturbation theory (CASPT2) results for the thymine nucleobase confirmed an accurate performance of the M06-2X method for predicting vertical and adiabatic ionization energies and AEAs.^44^

All geometry optimizations and frequency calculations were conducted for the gaseous phase using the Gaussian 09 program package.^45^ Structures and molecular orbitals were plotted with the GaussView 5.0 program.^46^

## 3 Results and Discussion

### 3.1 Experimental Results

**Figure 1:**
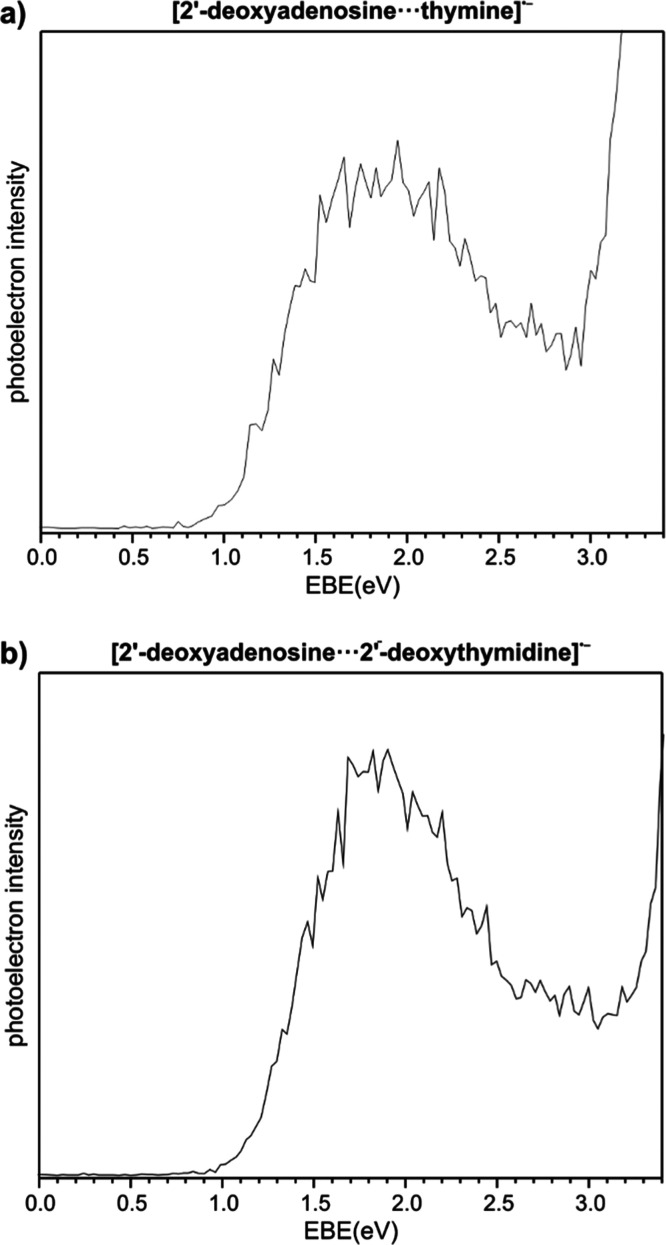
Photoelectron spectra of (a) 2′-deoxyadenosine···thymine (dAT^•–^) and (b) 2′-deoxyadenosine···2′-deoxythymidine (dAdT^•–^) [from ref (49)] recorded with 3.49 eV photons.

The photoelectron spectrum of dAT^•–^ recorded with 2.54 eV photons is shown in Figure 1a. The spectrum is dominated by a single broad peak at the EBE scale between ∼1.5 and 2.2 eV with the maximum at ∼1.9 eV. Such a signal is the result of a vertical photodetachment transition from the ground vibronic state of the 2′-deoxyadenosine···thymine anion radical to the ground vibronic state of its neutral counterpart. These EBE values correspond to the experimental VDE of the investigated heterodimer. Additionally, AEA as the EBE value at ∼10% of the rising signal may be extracted from the photoelectron spectrum. Thus, from the respective threshold of the signal in Figure 1a, the AEA for dAT^•–^ can be estimated to be ∼1.1 eV. The shape of the spectrum depicted in Figure 1, as well as the location of the signal at relatively high EBEs, is proof that the complex of the adenine nucleoside with thymine exists as a stable gaseous valence anion radical. Formation of dipole-bound type anions would manifest as a narrow signal at low EBEs, which reflects the situation where the excess electron is located outside the molecule being attracted by the positive pole of the molecule’s dipole.^47,48^

**Figure 2:**
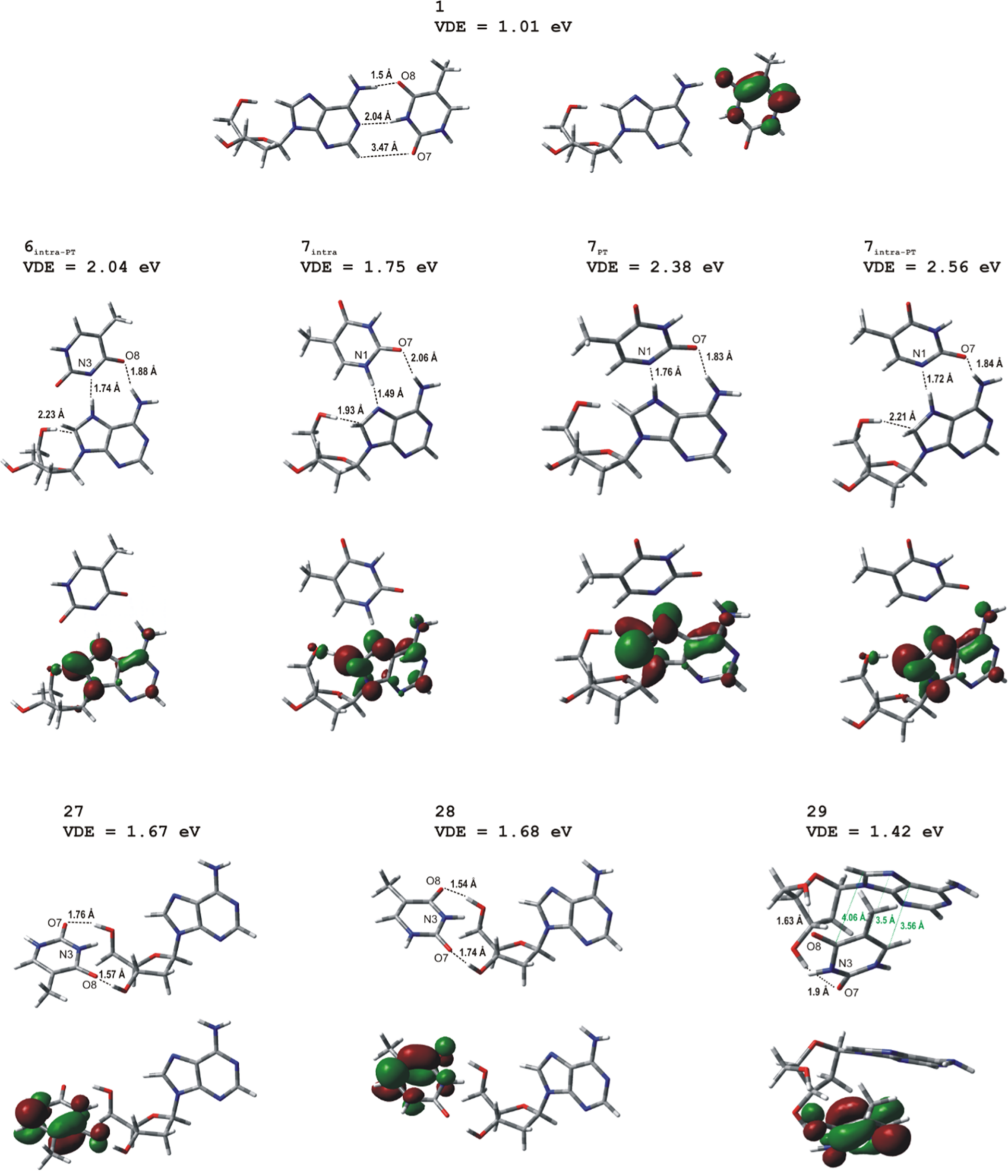
Structures and SOMOs of anion radical heterodimers dAT: three most stable dimers (**27**, **28**, and **29**) and the conformations of biological relevance, that is, Watson–Crick (**1**), Hoogsteen (**6**_**intra-PT**_), and reverse Hoogsteen (**7**_**intra-PT**_, **7**_**intra**_, and **7**_**PT**_). Structures are optimized at the M06-2X/6-31++G(d,p) level, and SOMOs are plotted with a contour value of 0.05*b*^–3/2^.

For the purpose of comparison, in addition to the photoelectron spectrum of 2′-deoxyadenosine···thymidine, we have included in Figure 1 the spectrum of 2′-deoxyadenosine···2′-deoxythymidine (dAdT^•–^) registered earlier with an identical experimental procedure.^49^ It may be noted that the signal maxima in both spectra are in the similar energy range, but the dAT^•–^ spectrum is noticeably broader. According to the M06-2X/6-31++G(d,p) calculations, the configuration of dAdT^•–^, responsible for the PES signal, is the complex stabilized by three intermolecular H-bonds. Namely, two hydrogen bonds connect hydroxyl groups of 2′-deoxyadenosine and thymine’s oxygens, (dA)3′OH···O7(dT) and (dA)5′OH···O8(dT), and the third H-bond exists between the adenine moiety and dT’s sugar, (dA)N1···3′OH(dT) (see Figure 2 for numbering). The latter bond is possible due to the parallel arrangement “head-to-tail” of the nucleosides. For this structure, the calculated AEA_G_ and VDE, 1.08 and 1.77 eV, respectively, reproduce well the experimental values extracted from the dAdT^•–^ photoelectron spectrum^49^ and, interestingly, would also fit the spectrum of the registered dAT^•–^. The similarity of both spectra (see Figure 1) combined with the information from the calculations about the geometry of the most stable dAdT^•–^ structure prompts the supposition that thymine is coordinated to the 2′-deoxyribose, utilizing its O7 and O8 atoms in the studied dAT^•–^ dimer.

### 3.2 Computational Results

#### 3.2.1 Search for the Dimer Responsible for the Shape of the Photoelectron Spectrum

To identify the geometry of dAT^•–^ responsible for the signal detected in Figure 1a, we have calculated possible geometrical configurations of 2′-deoxyadenosine and thymine. For the construction of dAT^•–^ dimers, we adopted the optimized at the B3LYP/DZP++ level geometries from the literature.^50^ The complexes were formed from the neutral 2′-deoxyadenosine and thymine radical anion, which is justified by a higher electron affinity of T when compared to dA.^51,52^ We have obtained 43 structures of dAT^•–^, and their relative thermodynamic stabilities, electron affinities, and VDEs are gathered in Table 1. Thermodynamic stabilities are expressed as the difference in the Gibbs free energies between the given structure and heterodimer **1**, in which nucleobases retain the Watson–Crick arrangement. Based on the type of interactions, these complexes can be categorized into seven groups. Geometries of all the complexes along with the corresponding singly occupied molecular orbitals (SOMOs) are presented in the Supporting Information.

**Table 1:** Relative Free-Energy Values (Δ*G*) with Respect to Dimer **1**, AEA_G_ of the Corresponding Neutrals, and VDE of the Anion Radical dAT Dimers Calculated at the M06-2X/6-31++G(d,p) Level^a^

no.	Δ*G*	AEA_E+E0_	AEA_G_	VDE
A···T				
**1**	0.00	0.21	0.29	1.01
**1**_**intra**_	8.46	–0.10	–0.08	0.84
**2**	4.96	0.01	0.08	0.74
**2**_**intra**_	8.59	–0.09	–0.08	0.84
**3**	3.19	0.04	0.11	0.72
**3**_**intra**_	1.14	0.20	0.19	1.13
**4**	5.78	0.07	0.12	0.76
**4**_**intra**_	12.54	–0.12	–0.18	0.78
**4**_**PT**_	3.26	0.21	0.22	1.35
**5**	4.07	0.13	0.18	0.82
**5**_**intra**_	11.27	–0.12	–0.13	0.76
**5**_**PT**_	3.44	0.20	0.21	1.36
**6**_**intra-PT**_	–1.94	0.32	0.35	2.04
**7**_**intra**_	–5.78	0.56	0.52	1.75
**7**_**PT**_	–8.49	0.58	0.63	2.38
**7**_**intra-PT**_	–9.95	0.67	0.70	2.56
**8**_**intra**_	–2.75	0.35	0.39	1.65
**9**_**intra**_	0.10	0.49	0.45	1.59
**10**_**intra**_	1.78	0.14	0.17	1.31
**11**_**intra**_	2.14	0.14	0.16	1.32
**12**	2.21	0.32	0.39	1.23
A···T/Sugar···T				
**13**	–5.71	0.69	0.71	1.42
**14**	–4.86	0.57	0.63	1.34
**15**	–9.14	0.74	0.75	1.42
**16**	–5.81	0.61	0.61	1.34
**17**	–6.35	0.45	0.49	1.27
**18**	–7.84	0.46	0.50	1.33
Sugar···T				
**19**	–11.63	0.73	0.86	1.44
**20**	–7.86	0.61	0.72	1.42
**21**	–8.11	0.78	0.76	1.46
**22**	–8.90	0.80	0.75	1.54
**23**	–9.94	0.89	0.89	1.53
**24**	–4.93	0.56	0.58	1.34
**25**	–5.28	0.36	0.46	1.26
Sugar···T/Sugar···T				
**26**	–11.69	0.95	0.99	1.66
**27**	–14.29	0.89	0.89	1.67
**28**	–14.79	0.91	0.91	1.68
Sugar···T/Sugar···T/Stack				
**29**	–12.66	0.79	0.79	1.42
Sugar···T/Stack				
**30**	–6.04	0.69	0.72	1.31
**31**	–6.99	0.42	0.49	0.94
**32**	–9.51	0.60	0.63	1.26
**33**	–5.89	0.44	0.48	1.34
Stack				
**34**	0.70	0.32	0.37	1.12

a Values of Δ*G* are given in kcal/mol and those of AEA_G_ and VDE in eV.

The first group comprises structures in which hydrogen bonds are formed between proton-donor and proton-acceptor sites of nucleobases (“A···T” family). This group is the largest and comprises 21 configurations. The structure denoted as **1**, where nucleobases are hydrogen bonded according to the canonical Watson–Crick scheme, belongs to this family. The second group, labeled “A···T/sugar···T”, contains six structures and their common feature is that thymine interacts with both adenine and sugar. The next family “sugar···T” consists of seven configurations, where thymine forms a single H-bond with 3′OH or 5′OH of dA. The family “sugar···T/sugar···T” comprises three complexes in which thymine is coordinated to the sugar moiety by two H-bonds. Structure **29** was assigned to a separate family “sugar···T/sugar···T/stack” because in addition to two H-bonds between the carbonyl oxygens of thymine and hydroxyl groups of the sugar, stacking interactions are present as the aromatic ring of thymine is parallel to the aromatic rings of adenine. Two remaining families, “sugar···T/stack” and “stack”, also involve complexes with monomers in the stacking arrangement. The bonding pattern in the family “sugar···T/stack” (four structures) is a combination of a single H-bond between 3′OH or 5′OH and O8 of thymine with dispersive interactions between aromatic systems of nucleobases. Finally, we have obtained one “stack” dAT^•–^ heterodimer structure **34** without any hydrogen bonds, where the cohesive force between monomers results mainly from dispersive interactions between the aromatic rings.

Based on the calculated Gibbs free energies of the anion radical complexes listed in Table 1, we have identified the most favorable spatial arrangement of thymine and 2′-deoxyadenosine within the anion radical heterodimer. In Figure 2 are shown the three most stable dimers (**27**, **28**, and **29**) and the conformations of biological relevance, that is Watson–Crick (**1**), Hoogsteen (**6**_**intra-PT**_), and reverse Hoogsteen (**7**_**intra-PT**_, **7**_**intra**_, and **7**_**PT**_). Thus, the thermodynamically most stable configuration is geometry **28**, consisting of thymine coordinated to the sugar of dA by two hydrogen bonds (dA)3′OH···O7(T) and (dA)5′OH···O8(T). The above observation is consistent with the previous PES/computational studies on dAdT^•–^ as such a bonding pattern was found also in the most stable anionic geometry of 2′-deoxyadenosine···2′-deoxythymidine.^49^ An analogous pattern of interactions in both complexes dAT^•–^ and dAdT^•–^ justifies to some extent high similarity of photoelectron spectra collated in Figure 1. However, there is a significant structural difference between the most stable configurations of dAdT^•–^ and dAT^•–^, that is, 2′-deoxythymidine in dAdT^•–^ forms an additional hydrogen bond between its 2′-deoxyribose and the adenine unit, (dA)N1···3′OH(dT). Obviously, such an interaction is impossible in the case of dAT^•–^ and as a result thymine in structure **28** is not aligned along the dA nucleoside like in dAdT^•–^. The simpler structure, and therefore the lack of certain interactions, is reflected by the slightly reduced tendency of **28** for the electron binding and the smaller stability of the resulting dAT^•–^ VB anion when compared to dAdT^•–^. Indeed, dimer **28** is characterized by AEA_G_ and VDE of 0.91 and 1.68 eV, respectively, whereas the corresponding values for dAdT^•–^ are 1.08 and 1.77 eV. Dimer **28** is by 14.8 kcal/mol more stable than structure **1**, where nucleobases interact according to the Watson–Crick scheme, and the AEA_G_ and VDE values calculated for **28** correspond well with the values extracted from the photoelectron spectrum shown in Figure 1a. Only 0.5 kcal/mol less stable than **28** is structure **27** from the same family “sugar···T/sugar···T”. The geometry of this heterodimer, as shown in Figure 2, is analogous to **28** because both thymines’ oxygens interact with both hydroxyl groups of 2′-deoxyribose. However, in structure **27**, thymine is flipped by 180° relative to its orientation in structure **28**, that is, in geometry **27** the (dA)3′OH···O8(T) and (dA)5′OH···O7(T) hydrogen bonds substitute (dA)3′OH···O7(T) and (dA)5′OH···O8(T) observed in structure **28**. Besides similar stabilities we have found for **27**practically identical to **28** AEA and VDE values 0.89 and 1.67 eV, respectively (see Table 1). The third structure in terms of thermodynamic stability is structure **29**, which is separated by only 2.13 kcal/mol on the Gibbs free-energy scale from the most stable **28**. This value suggests that geometry **29** can also contribute to the experimental PES spectrum. The calculated AEA_G_ and VDE for **29** are 0.79 and 1.42 eV. Thus, the presence of structure **29** under the experimental conditions could explain the broadening of the dAT spectrum compared to the dAdT one (cf. Figure 1a with 1b).

Complex **29** is the only representative of the family “sugar···T/sugar···T/stack”. Thymine in **29** is, like in **28**, coordinated to dA by two bonds, (dA)3′OH···O7(T) and (dA)5′OH···O8(T), and additionally is held by dispersive attraction. The presence of the stacking interactions results in an almost identical space arrangement of thymine relative to dA like in the above-mentioned dAdT^•–^. Also, the lengths of H-bonds in **29** are similar to those found in dAdT^•–^, namely, in **29** the lengths of (dA)3′OH···O7(T) and (dA)5′OH···O8(T) bonds are 1.9 and 1.63 Å, respectively, whereas in dAdT^•–^ the corresponding lengths are 1.89 and 1.62 Å (these distances are shorter in dimer **28**: 1.74 and 1.54 Å). When it comes to the stacking arrangement of nucleobases in dimer **29**, we have found that the atoms closest to each other are (T)(C5) and N7(A), which are separated by 3.5 Å. Thymine is not perfectly parallel to adenine’s ring, and this may be illustrated by the distance of ∼3.6 Å between atoms T(C6) and C5(A) and of ∼4.1 Å between T(C4) and C8(A). In Figure 2, we have marked the closest distances between nucleobases’ atoms.

AEA_G_ calculated for the next two most stable geometries, **26** and **19**, are 0.99 and 0.86 eV, while their VDEs are 1.66 and 1.44 eV, respectively. Heterodimer **26**, belonging to the “sugar···T/sugar···T” family, is characterized by the highest AEAs among the studied conformations. In this complex, thymine is coordinated to 3′OH of 2′-deoxyribose utilizing its O8, whereas the second interaction occurs between sugar’s hydroxyl group and endocyclic nitrogen of thymine: (dA)5′OH···N3(T) (see the Supporting Information). When it comes to complex **19**, only a single hydrogen bonding (dA)3′OH···O8(T) stabilizes the dimer. However, based on thermodynamic stability, the populations of the two heterodimers mentioned above, **26** and **19**, are probably negligible under the experimental conditions. Indeed, they are by 3.1 and 3.16 kcal/mol, respectively, less stable than the most stable complex **28**, which translates into the ratio of **26**/**19:28** less than 0.005 at *T* = 298 K.

Thus, one can come to the conclusion that mainly three conformers, **27**, **28**, and **29** (see Figure 2), are responsible for the shape of the photoelectron spectrum registered for the 2′-deoxyadenosine···thymine anion radical. These complexes are characterized by large and positive AEA_G_ values, (0.89, 0.91, and 0.79 eV) and their VDEs are predicted to be 1.67, 1.68, and 1.42 eV, respectively. Conformers **27**, **28**, and **29** have a common feature, namely, they contain two hydrogen bonds between carbonyl oxygen atoms of thymine and 2′-deoxyribose hydroxyl groups. The VDE values obtained for these structures, being in the range 1.4–1.7 eV, reproduce the experimental signal. Considering that fact that the signal is relatively broad in regard to the dAdT spectrum, the contribution of several conformers in the case of dAT seems to be probable. It is worth emphasizing that only one dimer geometry contributes to the PES spectrum of dAdT.^49^ Interestingly, we have found three heterodimers from the “A···T” family characterized by VDE values above 2 eV (see Figure 2 and the Supporting Information). These are **6**_**intra-PT**_ (VDE = 2.04 eV), **7**_**PT**_ (VDE = 2.38 eV), and **7**_**intra-PT**_ (VDE = 2.56 eV). The last structure is the most stable among them, being 4.8 kcal/mol less stable relative to **28**. Such a large difference in stability is, however, too large to allow dimer **7**_**intra-PT**_ to be populated in the experiment, and this conclusion is further supported by the fact that the spectrum shown in Figure 1 does not exhibit any peak near 2.5 eV at the EBE scale.

#### 3.2.2 Excess Electron Localization in the dAT Dimer

The SOMO shape of the most stable anionic configurations, **27**, **28**, and **29**, reveals that in each case the excess electron resides on the π* orbital of thymine. Therefore, it can be said that the interaction of thymine with 2′-deoxyadenosine plays a stabilizing role for the thymine anion radical, which when isolated is very unlikely to appear as a VB anion.^47,48,53^ The stabilization of T^•–^ by 2′-deoxyadenosine, as reflected by VDE, is of similar magnitude as the stabilization of T^•–^ by three and five water molecules for which Kim et al. calculated VDEs at the B3LYP/DZP++ level to be 1.46 eV (T···(H_2_O)_3_) and 1.6 eV (T···(H_2_O)_5_).^54^

Among scrutinized combinations of monomers, we have also identified many dAT^•–^ complexes where 2′-deoxyadenosine is the host of the excess electron. Such a charge distribution has not been observed so far in two-component anion radical complexes involving adenine and thymine. All these 15 structures belong to the “A···T” group which comprises a total of 21 structures (in the remaining families, the excess electron is located exclusively on thymine). Generally, the complexes in the “A···T” family are stabilized by both inter- and intramolecular interactions, and depending on the arrangement of monomers and intrinsic conformation of dA, the excess electron localizes on thymine or adenine. Delocalization over both monomers is not observed. The attachment of the excess electron to adenine is promoted by a specific nucleoside conformation, where the hydroxyl group of the sugar is hydrogen bonded to the C8 atom of adenine (5′OH···C8). Dimers involving such an intramolecular 5′OH···C8 bond within the nucleoside are labeled by the subscript “intra”. We have identified several complexes which can exist in two variants, one with 2′-deoxyadenosine possessing intramolecular H-bonding and the second, where such a 5′OH···C8 bond does not occur. In these configurations, thymine is coordinated by three or two H-bonds to adenine’s N6H/N1/C2H or C2H/N3 centers. Geometries of the above dimers are visualized in the Supporting Information, and the corresponding pairs are labeled as follows: **1** and **1**_**intra**_, **2** and **2**_**intra**_, **3** and **3**_**intra**_, **4** and **4**_**intra**_, and **5** and **5**_**intra**_. Thus, in all the “intra” structures **1**_**intra**_, **2**_**intra**_, **3**_**intra**_, **4**_**intra**_, and **5**_**intra**_, the SOMO is localized on the adenine moiety. In contrast, the absence of an intramolecular hydrogen bond in dA causes the electron to localize on thymine (dimers **1**, **2**, **3**, **4**, and **5**). Distribution of the negative charge over the adenine’s framework in the “intra” dimers makes such anion radicals considerably less stable, in terms of Gibbs free energy, when compared to the “non-intra” structure with a negative charge on thymine. Exceptions are **3** and **3**_**intra**_, where the “intra” geometry is slightly more stable than the corresponding “non-intra” one (by 2 kcal/mol).

Additionally, we have identified five structures (**4**_**PT**_, **5**_**PT**_, **6**_**intra-PT**_, **7**_**PT**_, and **7**_**intra-PT**_) with an electron attachment-induced PT to adenine. The SOMOs of these PT complexes demonstrate that the excess electron is localized to adenine (see the Supporting Information). Interestingly, dimers **6**_**intra-PT**_ and **7**_**intra-PT**_ feature both phenomena, that is intramolecular 5′OH···C8 bond within dA and PT (here it should be noted that **7**_**intra-PT**_ is characterized by the highest thermodynamic stability within the “A···T” family and the highest VDE value among all the investigated complexes, 2.56 eV). From the relative thermodynamic stability of the compounds (values of Δ*G* in Table 1), as well as from the VDE values, it can be concluded that PT from N1(T) to N7(dA) is the most favorable and occurs in **7**_**PT**_ and **7**_**intra-PT**_.

Although the dimeric anions in which the electron is localized on adenine have no contribution to the measured PES spectrum (due to their relative stability; see Table 1), they might be important in a biological environment, that is, under the geometrical constraints of DNA. Indeed, we have found five dimers which are more stable than the canonical Watson–Crick structure of AT—the hallmark of DNA. Four of these structures adopt the biologically important Hoogsteen^55,56^ (**6**_**intra-PT**_) and reverse Hoogsteen^57,58^ (**7**_**intra-PT**_, **7**_**intra**_ and **7**_**PT**_) conformations, and electron attachment leads to PT from thymine to adenine. As a consequence, PT, resulting in the neutralization of the negative charge localized on adenine, prevents further migration of the electron to the backbone and eventually the strand cleavage.

It is worth emphasizing that a spontaneous protonation of adenine upon the excess electron attachment to its π* orbital was observed in our earlier combined PES/computational studies on adenine···formic acid (A···FA) and 9-methyladenine···formic acid (9MA···FA) complexes.^59^ In the dimers, A···FA and 9MA···FA, the preferred site for protonation in adenine by the OH group of formic acid is N3 and the second is N7. The one less prone to protonation is the N1 site of adenine, involved in the WC pairing scheme in DNA. We have also studied the photoelectron spectra of 1:2 and 1:3 complexes, A···2FA, 9MA···2FA, and A···3FA.^60^ In all the considered trimers and tetramers, attachment of an electron led to barrierless double PT from the formic acid molecules. A favorable protonation of the N3 and N7 sites of A, observed in the present project and in the past, remains in agreement with the results of MD simulations performed by McAllister et al. for nucleotides immersed in water.^11^ These authors have found that the N3 site of adenine in the nucleotide protonates spontaneously and the second hydrogen bond forms between water and N7 of adenine. McAllister et al.^11^ and also Smyth and Kohanoff^13^ emphasized the effect of the PT phenomenon involving nucleobases on the kinetics of the DNA strand-breaking reaction upon excess electron attachment.

## 4 Conclusions

The results of our experimental computational study can be summarized in the following points:

(1) Stable VB anion radical dimers of 2′-deoxyadenosine···thymine, dAT^•–^, resulting from the attachment of LEEs to the gaseous mixture of neutral dimers, are formed under experimental conditions. The registered PES spectrum is typical for the VB anion and features a single broad peak at the EBE scale between ∼1.5 and 2.2 eV with the maximum at ∼1.9 eV, which corresponds to the experimental VDE. The measured value of AEA for dAT is estimated to be ∼1.1 eV.
(2) In the computational part of the project, we have identified the structures of anion radical dimers which most probably are formed during the experiment. The most stable dimeric anion (structure **28**) is by almost 15 kcal/mol more stable than the structure, where nucleobases interact according to the Watson–Crick scheme (structure **1**). In configuration **28**, thymine is coordinated to the sugar of dA by two hydrogen bonds (dA)3′OH···O7(T) and (dA)5′OH···O8(T). The calculated AEA_G_ and VDE of 0.91 and 1.68 eV, respectively, correspond well to the values extracted from the photoelectron spectrum. Additionally, the presence of two other less stable configurations in the equilibrated mixture of anions cannot be ruled out (**27** and **29**). In both structures, thymine’s oxygens interact with both hydroxyl groups of 2′-deoxyribose. These complexes are characterized by large and positive AEA_G_ values of 0.89 and 0.79 eV, and their VDEs are predicted to be 1.67 and 1.42 eV, respectively. Thus, the occurrence of these structures under the experimental conditions could explain the broadening of the PES signal with regard to that of dAdT.
(3) Conformational search performed in the present study also yielded several anionic structures, more stable than the Watson–Crick configuration, in which the excess electron is localized on adenine. These configurations correspond to the biologically relevant Hoogsteen and reverse Hoogsteen arrangements; hence, such anions may form in DNA. Electron attachment to these dimers triggers PT to adenine thus preventing a LEE-induced strand cleavage.

## Supporting Information Available

Large-scale visualization of the optimized structures of 43 anion radical 2′-deoxyadenosine···thymine dimers, their SOMOs plotted with a contour value of 0.05*b*^–3/2^, and complete refs (33) and (45) (PDF)
