# Supplementary material for: Electrophilic Properties of 2′-Deoxyadenosine···Thymine Dimer: Photoelectron Spectroscopy and DFT Studies
Source: J Phys Chem A. 2021 Jul 26;125(30):6591–9. doi: 10.1021/acs.jpca.1c03803 (PMC8389985; doi:10.1021/acs.jpca.1c03803)
Supplement: Supplementary file 1 — jp1c03803_si_001.pdf [file jp1c03803_si_001.pdf]

Supporting Information

Electrophilic Properties of 2'-Deoxyadenosine···Thymine Dimer: Photoelectron Spectroscopy and DFT Studies

Piotr Storoniak,[a] Janusz Rak,[a] Haopeng Wang,[b] Yeon Jae Ko[b] and Kit H. Bowen[b]*

[a] Faculty of Chemistry, University of Gdańsk, Wita Stwosza 63, 80-308 Gdańsk, Poland.

[b] Department of Chemistry, Johns Hopkins University, Baltimore, Maryland 21218, United States.

E-mail: piotr.storoniak@ug.edu.pl

Table of Contents

Large scale visualization of the optimized structures of 43 anion radical 2’-deoxyadenosine···thymine dimers and their singly occupied molecular orbitals plotted with a contour value of 0.05 $b^{-3/2}$.

Complete Reference 33 and 45.

1
VDE = 1.01 eV

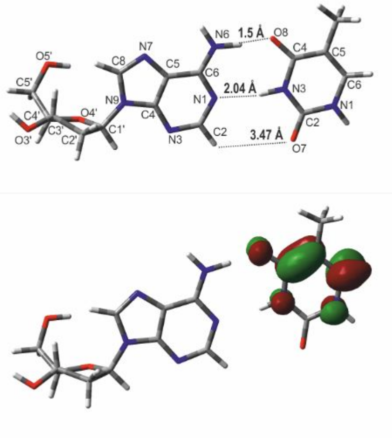

1_{intra}
VDE = 0.84 eV

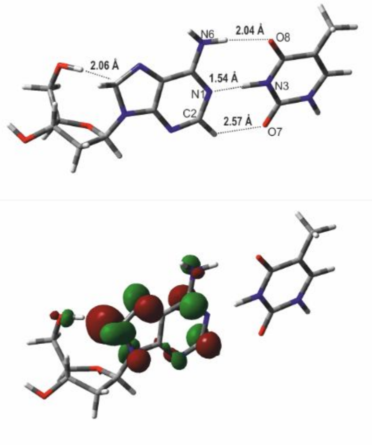

2
VDE = 0.74 eV

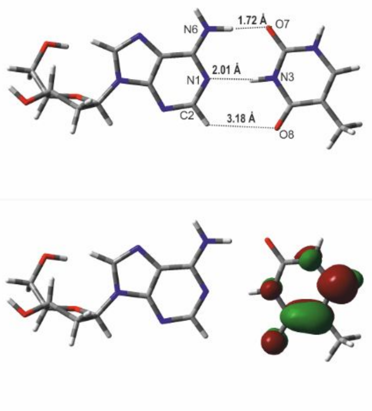

2_{intra}
VDE = 0.84 eV

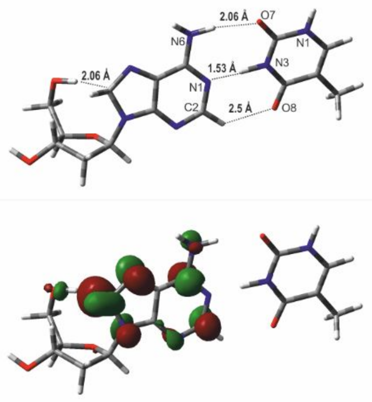

3
VDE = 0.72 eV

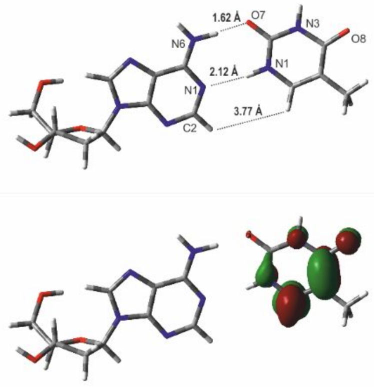

3_{intra}
VDE = 1.13 eV

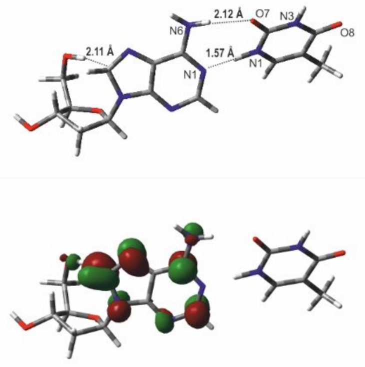

A···T (2/5)

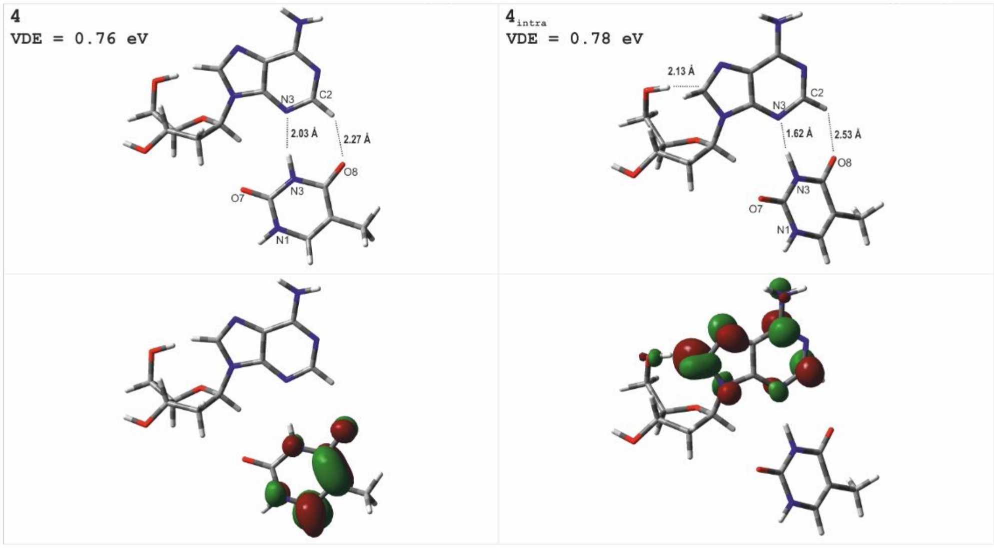
4
VDE = 0.76 eV
N3
C2
2.03 Å
2.27 Å
O8
O7
N3
N1
4intra
VDE = 0.78 eV
2.13 Å
N3
C2
1.62 Å
2.53 Å
O8
O7
N3
N1

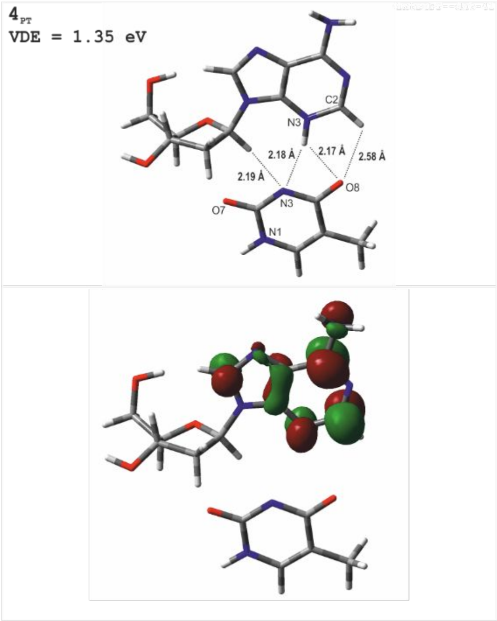
4PT
VDE = 1.35 eV
C2
N3
2.18 Å
2.17 Å
2.58 Å
2.19 Å
N3
O8
O7
N1

A···T (3/5)

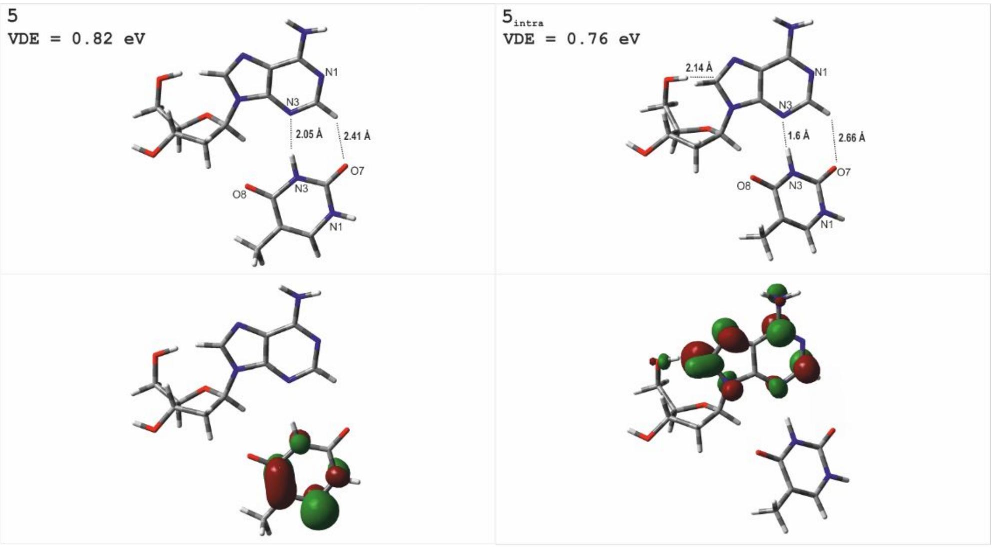
5
VDE = 0.82 eV
N1
N3
2.05 Å
2.41 Å
O7
O8
N3
N1
5_{intra}
VDE = 0.76 eV
2.14 Å
N1
N3
1.6 Å
2.66 Å
O7
O8
N3
N1

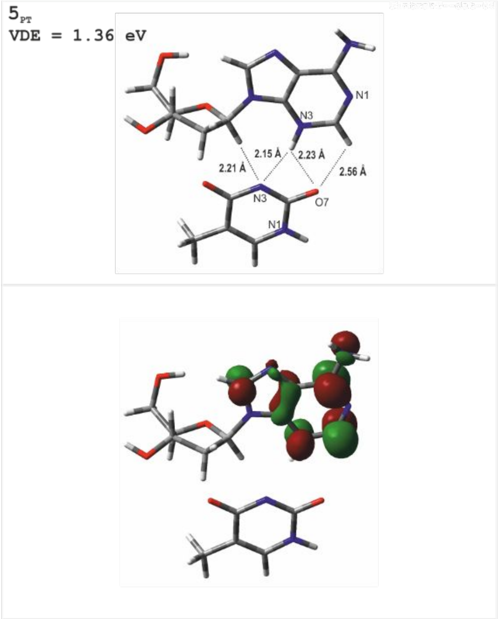
5_{PT}
VDE = 1.36 eV
N1
N3
2.15 Å
2.23 Å
2.21 Å
2.56 Å
N3
O7
N1

A···T (4/5)

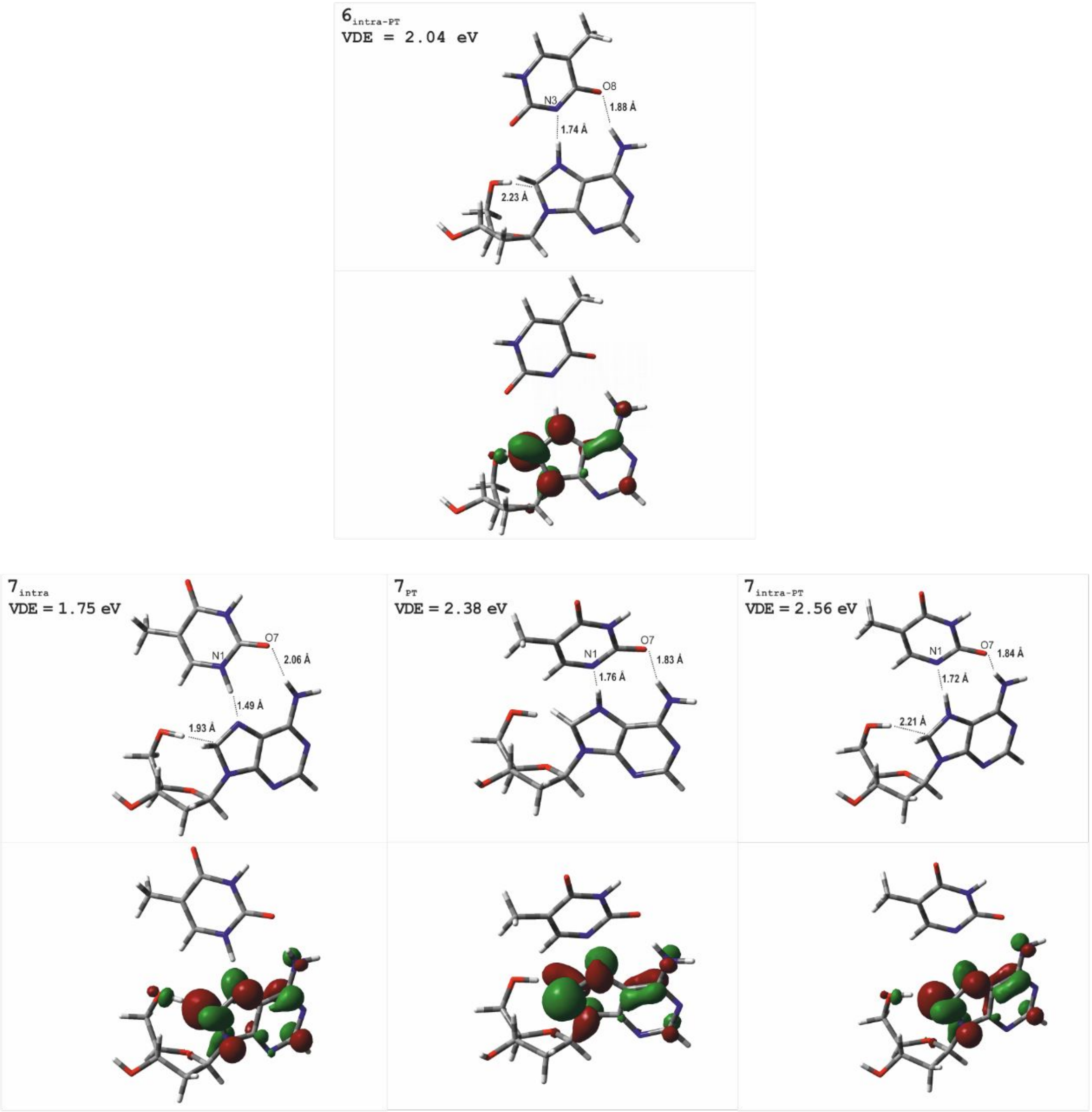

6intra-PT
VDE = 2.04 eV
N3
O8
1.88 Å
1.74 Å
2.23 Å
7intra
VDE = 1.75 eV
N1
O7
2.06 Å
1.49 Å
1.93 Å
7PT
VDE = 2.38 eV
N1
O7
1.83 Å
1.76 Å
7intra-PT
VDE = 2.56 eV
N1
O7
1.84 Å
1.72 Å
2.21 Å

A···T (5/5)

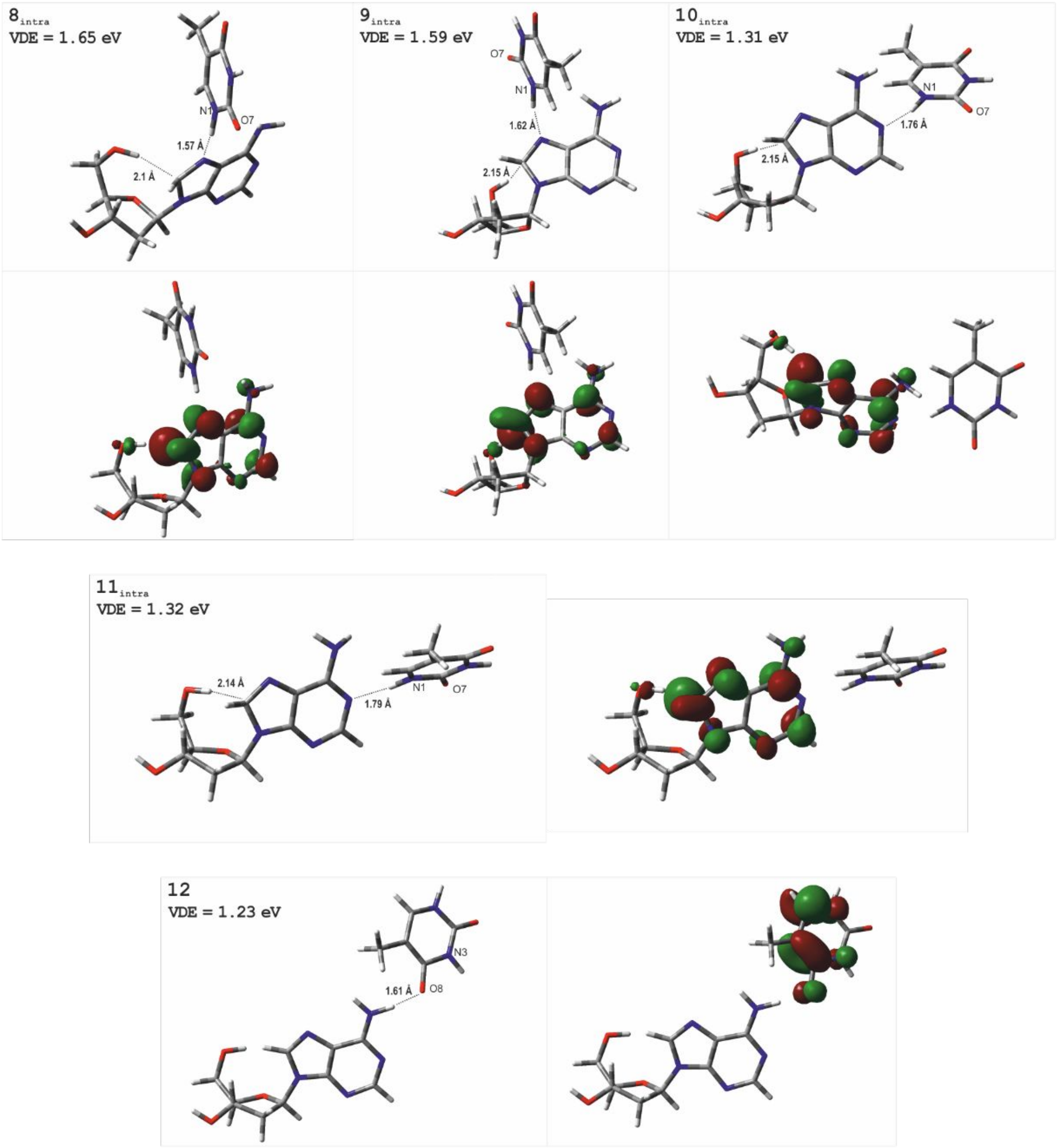

8intra
VDE = 1.65 eV
N1
O7
1.57 Å
2.1 Å
9intra
VDE = 1.59 eV
O7
N1
1.62 Å
2.15 Å
10intra
VDE = 1.31 eV
N1
O7
1.76 Å
2.15 Å
11intra
VDE = 1.32 eV
2.14 Å
N1
O7
1.79 Å
12
VDE = 1.23 eV
N3
1.61 Å
O8

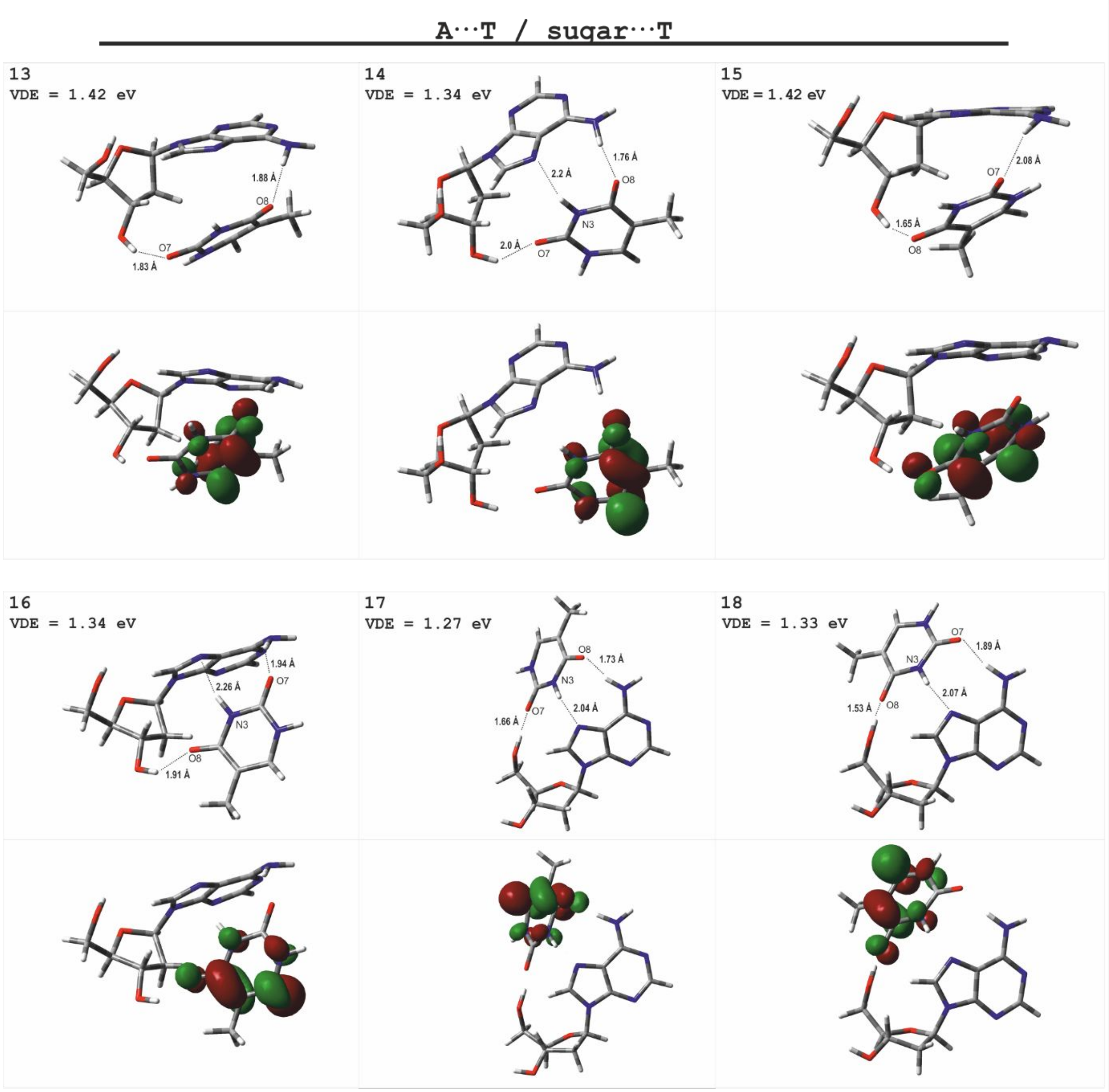
A···T / sugar···T
13
VDE = 1.42 eV
1.88 Å
O8
O7
1.83 Å
14
VDE = 1.34 eV
1.76 Å
2.2 Å
O8
N3
2.0 Å
O7
15
VDE = 1.42 eV
O7
2.08 Å
1.65 Å
O8
16
VDE = 1.34 eV
1.94 Å
O7
2.26 Å
N3
O8
1.91 Å
17
VDE = 1.27 eV
O8
1.73 Å
N3
2.04 Å
1.66 Å
O7
18
VDE = 1.33 eV
O7
1.89 Å
N3
2.07 Å
1.53 Å
O8

sugar···T

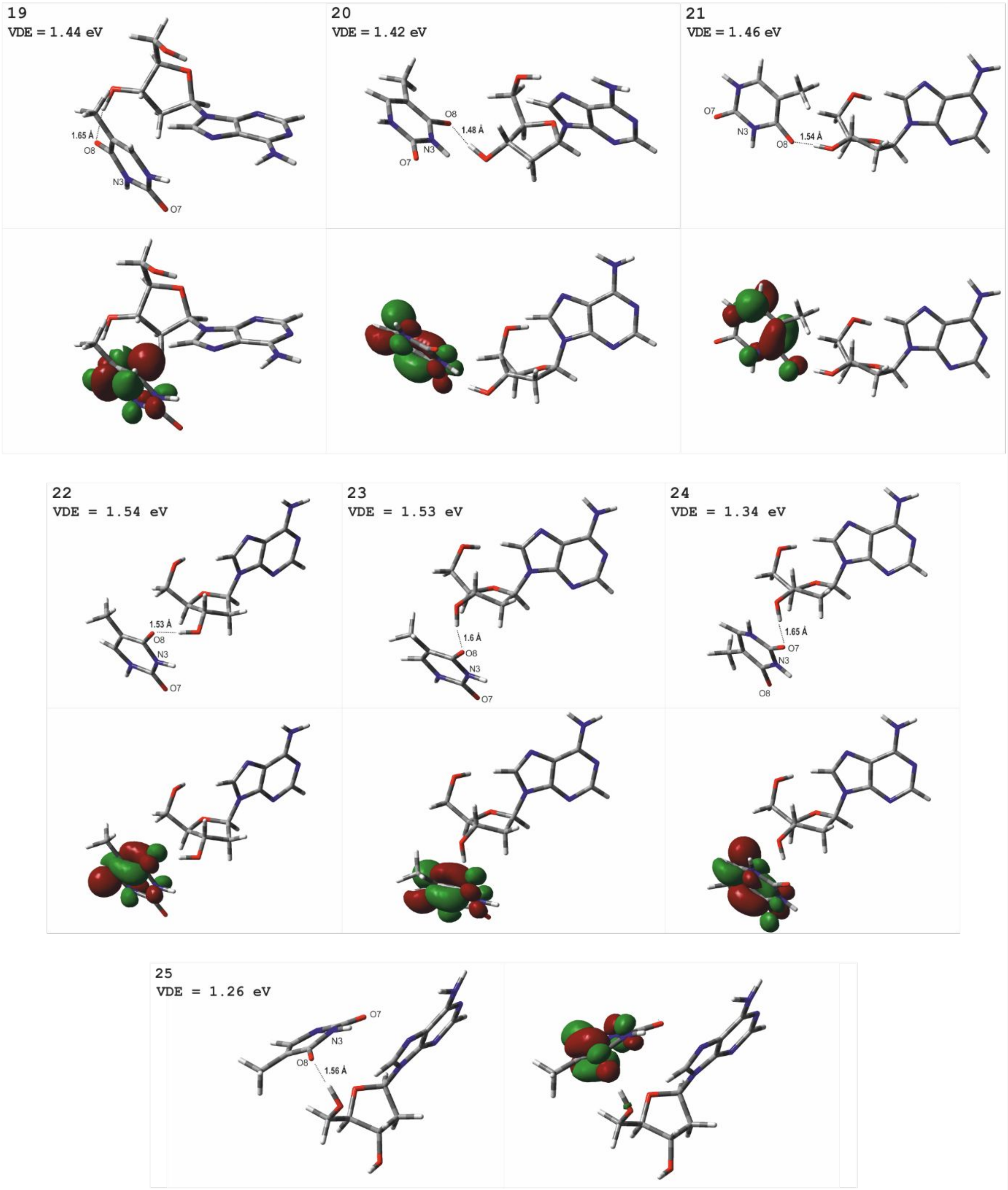
19
VDE = 1.44 eV
20
VDE = 1.42 eV
21
VDE = 1.46 eV
22
VDE = 1.54 eV
23
VDE = 1.53 eV
24
VDE = 1.34 eV
25
VDE = 1.26 eV

sugar···T / sugar···T

26
VDE = 1.66 eV

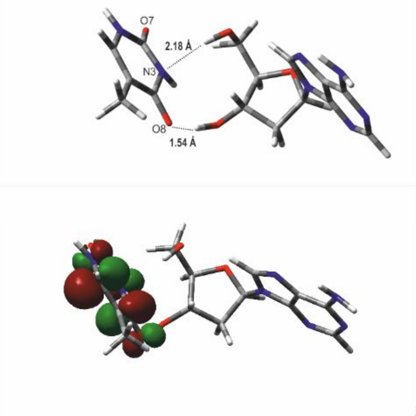

27
VDE = 1.67 eV

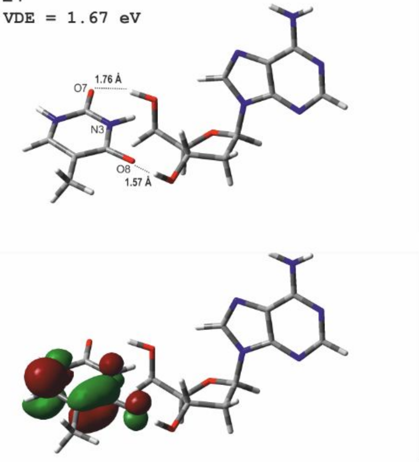

28
VDE = 1.68 eV

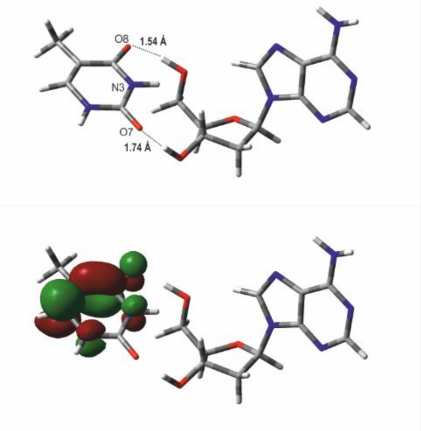

sugar···T / sugar···T / stack

29
VDE = 1.42 eV

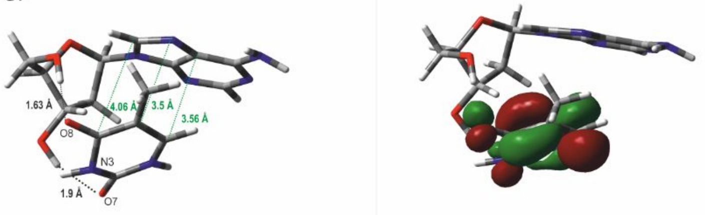

sugar···T / stack

30
VDE = 1.31 eV

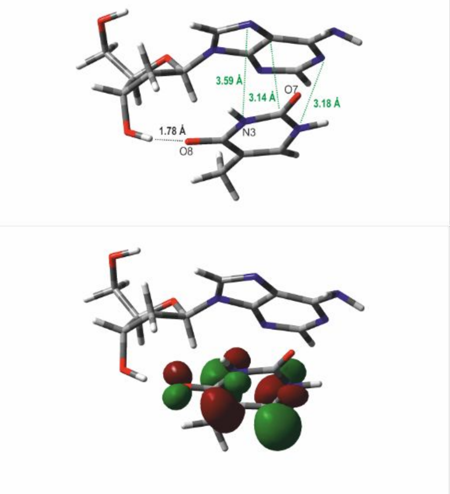

31
VDE = 0.94 eV

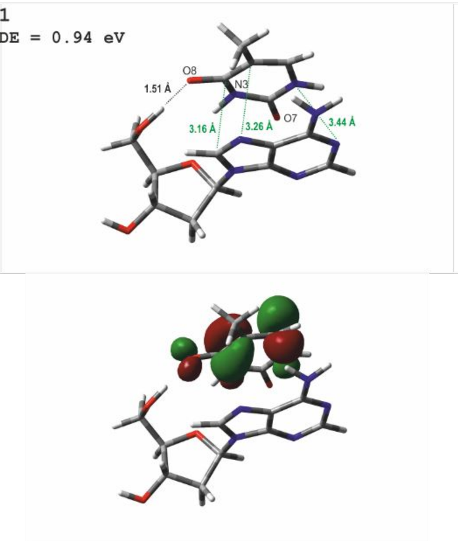

32
VDE = 1.26 eV

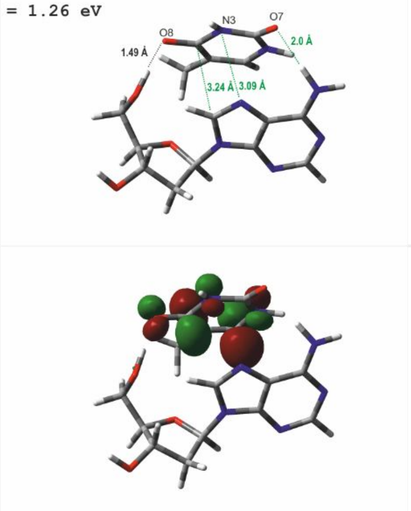

33
VDE = 1.34 eV

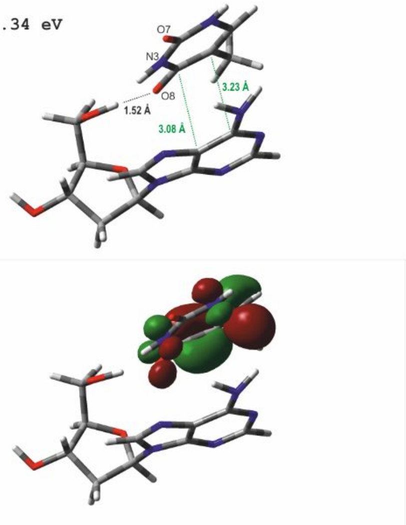

stack

34
VDE = 1.12 eV

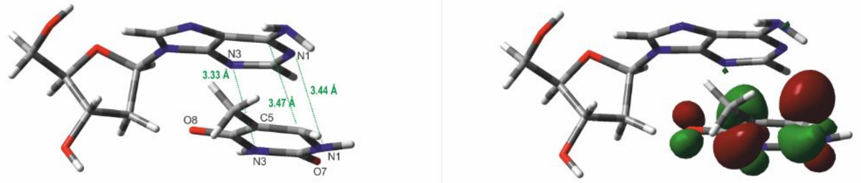

Complete Reference 33:

Rak, J.; Mazurkiewicz, K.; Kobyłecka, M.; Storoniak, P.; Haranczyk, M.; Dąbkowska, I.; Bachorz, R. A.; Gutowski, M.; Radisic, D.; Stokes, S. T.; Eustis, S. N.; Wang, D.; Li, X.; Ko, Y. J.; Bowen, K. H. Stable Valence Anions of Nucleic Acid Bases and DNA Strand Breaks Induced by Low Energy Electrons. In Radiation Induced Molecular Phenomena in Nucleic Acid: A Comprehensive Theoretical and Experimental Analysis; Shukla, M., Leszczynski, J., Eds.; Challenges and Advances in Computational Chemistry and Physics; Springer: Dordrecht, the Netherlands, 2008; pp 619−667.

Complete Reference 45:

Frisch, M. J.; Trucks, G. W.; Schlegel, H. B.; Scuseria, G. E.; Robb, M. A.; Cheeseman, J. R.; Scalmani, G.; Barone, V.; Mennucci, B.; Petersson, G. A.; Nakatsuji, H.; Caricato, M.; Li, X.; Hratchian, H. P.; Izmaylov, A. F.; Bloino, J.; Zheng, G.; Sonnenberg, J. L.; Hada, M.; Ehara, M.; Toyota, K.; Fukuda, R.; Hasegawa, J.; Ishida, M.; Nakajima, T.; Honda, Y.; Kitao, O.; Nakai, H.; Vreven, T.; Montgomery, Jr. J. A.; Peralta, J. E.; Ogliaro, F.; Bearpark, M.; Heyd, J. J.; Brothers, E.; Kudin, K. N.; Staroverov, V. N.; Keith, T.; Kobayashi, R.; Normand, J.; Raghavachari, K.; Rendell, A.; Burant, J. C.; Iyengar, S. S.; Tomasi, J.; Cossi, M.; Rega, N.; Millam, J. M.; Klene, M.; Knox, J. E.; Cross, J. B.; Bakken, V.; Adamo, C.; Jaramillo, J.; Gomperts, R.; Stratmann, R. E.; Yazyev, O.; Austin, A. J.; Cammi, R.; Pomelli, C.; Ochterski, J. W.; Martin, R. L.; Morokuma, K.; Zakrzewski, V. G.; Voth, G. A.; Salvador, P.; Dannenberg, J. J.; Dapprich, S.; Daniels, A. D.; Farkas, O.; Foresman, J. B.; Ortiz, J. V.; Cioslowski, J., Fox, D. J. Gaussian 09, Revision E.01, Gaussian, Inc., Wallingford CT, 2013.
